# High-intensity statin therapy yields better outcomes in acute coronary syndrome patients: a meta-analysis involving 26,497 patients

**DOI:** 10.1186/s12944-020-01369-6

**Published:** 2020-08-23

**Authors:** Shiyong Yu, Jun Jin, Zhongxiu Chen, Xiaolu Luo

**Affiliations:** 1 Institute of Cardiovascular Diseases, Xinqiao Hospital, Army Medical University (Third Military Medical University), Chongqing, China; 2 Department of Cardiology, West China Hospital, Sichuan University, Chengdu, China; 3 HuoCheNan Community Health Service Center, Wuhou District, Chengdu, 610041 China

**Keywords:** High-intensity statin therapy, Acute coronary syndromes, Efficacy, Safety, Major adverse cardiovascular events, Asians

## Abstract

**Background:**

Whether high-intensity statin treatment provides more clinical benefits compared with standard statin regimens in acute coronary syndrome (ACS) patients remains controversial. This meta-analysis aimed to comparatively assess high-intensity and standard statin regimens for efficacy and safety in patients with ACS.

**Methods:**

The PubMed, EMBASE, and Cochrane Library databases were searched for studies assessing high-intensity vs. standard statin regimens for ACS treatment from inception to April 2020. The publication language was limited to English, and 16 randomized controlled trials were finally included in this study, with a total of 26,497 patients.

**Results:**

Compared to the standard statin regimens, the relative ratio (RR) of major adverse cardiovascular events (MACE) in ACS patients treated by high-intensity statin was 0.77 (95%CI, 0.68–0.86; *P* < 0.00001; prediction interval, 0.56–1.07). In subgroup analysis, high-intensity statin therapy resulted in more clinical benefits regarding MACE compared with standard statin treatment in both Asian (RR = 0.77; 95%CI, 0.61–0.98; *P* = 0.03) and non-Asian (RR = 0.79; 95%CI, 0.71–0.89; *P* < 0.0001) patients. Although adverse events were acceptable in patients with ACS administered high-intensity statin therapy, this treatment was associated with a higher rate of adverse events (4.99% vs. 2.98%), including myopathy/myalgia and elevated liver enzymes, as reflected by elevated serum aminotransferase or aminotransferase amounts.

**Conclusion:**

The current findings indicated that high-intensity statin therapy might be beneficial in patients with ACS, and close monitoring for adverse effects should be performed.

## Introduction

Numerous studies have shown that 3-hydroxy-3-methylglutaryl-coenzyme A reductase inhibitors (statins) reduce the risk of death and cardiovascular events in acute coronary syndrome (ACS) cases [1, 2]. Based on the MIRACL study, which evaluated the effects of atorvastatin at 80 mg versus (vs.) placebo, and the Prove-it trial, which comparatively evaluated atorvastatin (80 mg) and pravastatin (40 mg), the American College of Cardiology recommended high-intensity statin treatment for ST-elevation myocardial infarction (MI) management in ACS cases, with a level of evidence of B [3]. Noticeably, the Prove-it trial showed an overt reduction of major adverse cardiovascular events (MACE) with high-intensity treatment and an elevated risk of adverse events [1]. Nevertheless, whether high-intensity statin treatment confers more clinical benefits compared with standard statin therapy in patients with ACS remains controversial, although the previous Prove-it trial indicated high-intensity statin therapy further reduces MACE [4]. A previous meta-analysis showed that intensive statin administration benefits more than standard statin treatment in the prevention of non-fatal cardiovascular events, such as stroke, by decreasing cardiovascular mortality in individuals with stable coronary heart disease (18,889 patients) or ACS (8659 patients) [5]. Recently, studies that were not included in the latter meta-analysis reported discrepant findings, claiming that high-intensity statin treatment has no significant effect on MACE reduction in ACS cases in comparison with standard statin administration [6, 7].

Because of the above controversial findings, the present meta-analysis of randomized controlled trials (RCTs) was performed to comparatively evaluate high-intensity and standard statin regimens for efficacy and safety in patients with ACS.

## Methods

### Data source and search strategy

The PubMed, EMBASE, and Cochrane Library databases were searched for relevant English publications from inception to April 2020. The search was performed with “hydroxymethylglutaryl-CoA reductase inhibitors”, “myocardial infarction”, “unstable angina”, “acute coronary syndrome” as medical subject headings (MeSH), in addition to the key word statin. Search results were limited to RCTs, including adult populations (age ≥ 18 years). The reference lists of the retrieved reports were manually searched for potential additional eligible studies.

### Study eligibility

Two investigators (Yu and Chen) searched the databases in an independent fashion. A third reviewer (Luo) settled any discrepancies. First, duplicate articles were removed. Then, the titles and abstracts of the retained publications were screened before full-text retrieval. Meeting abstracts, editorials, and reviews were also excluded from the present analysis. High-intensity statin was defined as atorvastatin administered at 40–80 mg, rosuvastatin at 20–40 mg, or simvastatin at 80 mg, i.e., medication amounts higher than the standard doses described in recent guidelines [8, 9]. The inclusion criteria in the meta-analysis were: (1) RCT involving patients with a diagnosis of ACS; (2) comparison of high-intensity statin and standard-dose; (3) follow-up of at least 1 month; (4) primary endpoint as the combined outcome of myocardial infarction (MI), stroke and death (or MACE), defined by individual investigators.

### Data extraction and quality evaluation

Two investigators (Yu and Luo) performed data extraction from the eligible studies in an independent fashion. Disagreement was resolved by discussion until consensus. After identifying all the relevant full articles, the characteristics of the included studies were extracted. The primary endpoint was the combined outcome of MI, stroke, and death (or MACE), defined by the individual investigators. The secondary individual endpoints were MI, stroke, cardiovascular death, and total death. Zhou et al. [10] was referred to for calculating the corresponding HR of the missing data. Kaplan-Meier curve was read by using GetData Graph Digitizer 2.26 unless the adequate data could be extracted.

Two reviewers assessed the risk of bias in each trial independently, using the assessment tool and strictly following the protocol from the Cochrane Handbook [11].

### Outcomes

The primary outcome was the combined outcome of MI, stroke, and death (i.e., MACE), defined by the individual investigators. The secondary individual outcomes were MI, stroke, cardiovascular death, and total death.

### Statistical analysis

The hazard ratio (HR) or relative risk (RR) and 95% confidence interval (CI) for each endpoint in the high-intensity and standard-dose groups were determined. Cochrane Q test and the I^2^ statistic were determined to assess heterogeneity, with *P* < 0.1 or I^2^ > 50% indicating significant heterogeneity. In the case of heterogeneity, a random-effects model was applied for analysis; otherwise, a fixed-effects model was used [12]. Two-sided *P* < 0.05 was considered statistically significant. Subgroup analyses were based on statin treatment duration and race. Sensitivity analysis was performed by the sequential exclusion of individual trials, whose effects on the overall findings were assessed. For the meta-regression analysis, univariable random-effect meta-regression analyses were conducted; multivariable meta-regression analyses were performed if the univariable analyses were significant. The Egger’s test and funnel plot analysis were performed to assess publication bias in case more than 10 studies were involved in the meta-analysis or subgroup analyses. *P*_Egger_ < 0.05 was considered to indicate publication bias. Review Manager 5.3 (the Cochrane Collaboration; http://ims.cochrane.org/revman) and R 4.0.0 and *meta* package were used for data analysis. The GRADE methods involve the risk of bias, the directness of evidence, heterogeneity of data, the precision of effect estimates, and risk of publication bias in order to provide a measure of confidence about the correctness of the estimates [13]. The GRADEpro software was used for the GRADE analysis. The prediction intervals were estimated for random-effect models according to the methods by IntHout et al. [14]. Briefly, a 95% prediction interval is an estimate of where the actual effects should be expected for 95% of comparable trials, which might be performed in the future. Thus, it could help assess the variability of an intervention’s effects in various settings. When there is no between-study heterogeneity, the prediction and corresponding confidence intervals coincide. However, heterogeneity results in the prediction interval covering a broader range than the confidence interval. In case of a statistically significant effect with the totality of 95%CIs on the same side of the null, the corresponding 95% prediction interval might indicate the possibility of values being on both sides of the null. In this case, conclusions based on CIs may be erroneous.

The predefined analyses were the impact of high-intensity statins on MACE; the impact of high-intensity statins on MACE in Asians and non-Asians; the impact of high-intensity statins on MACE stratified by the time to randomization; the impact of high-intensity statins on individuals MACEs; the impact of high-intensity statins on lipids; and adverse events. The post hoc analyses were sensitivity analysis for simvastatin 80 mg, evaluation of the fixed-effect model for the primary outcome, and meta-regression for a causal relationship between LDL-C and MACE. Previous studies have shown that early statin administration for 4 months decreases mortality and cardiovascular events [15]. Aiming to more reliably estimate the time-course effects of high-intensity statin treatment in ACS, high-intensity and standard statin regimens were assessed with stratification by time from randomization (0–1 month, 1–12 months, and ≥ 12 months).

## Results

### Literature search

**Fig. 1:**
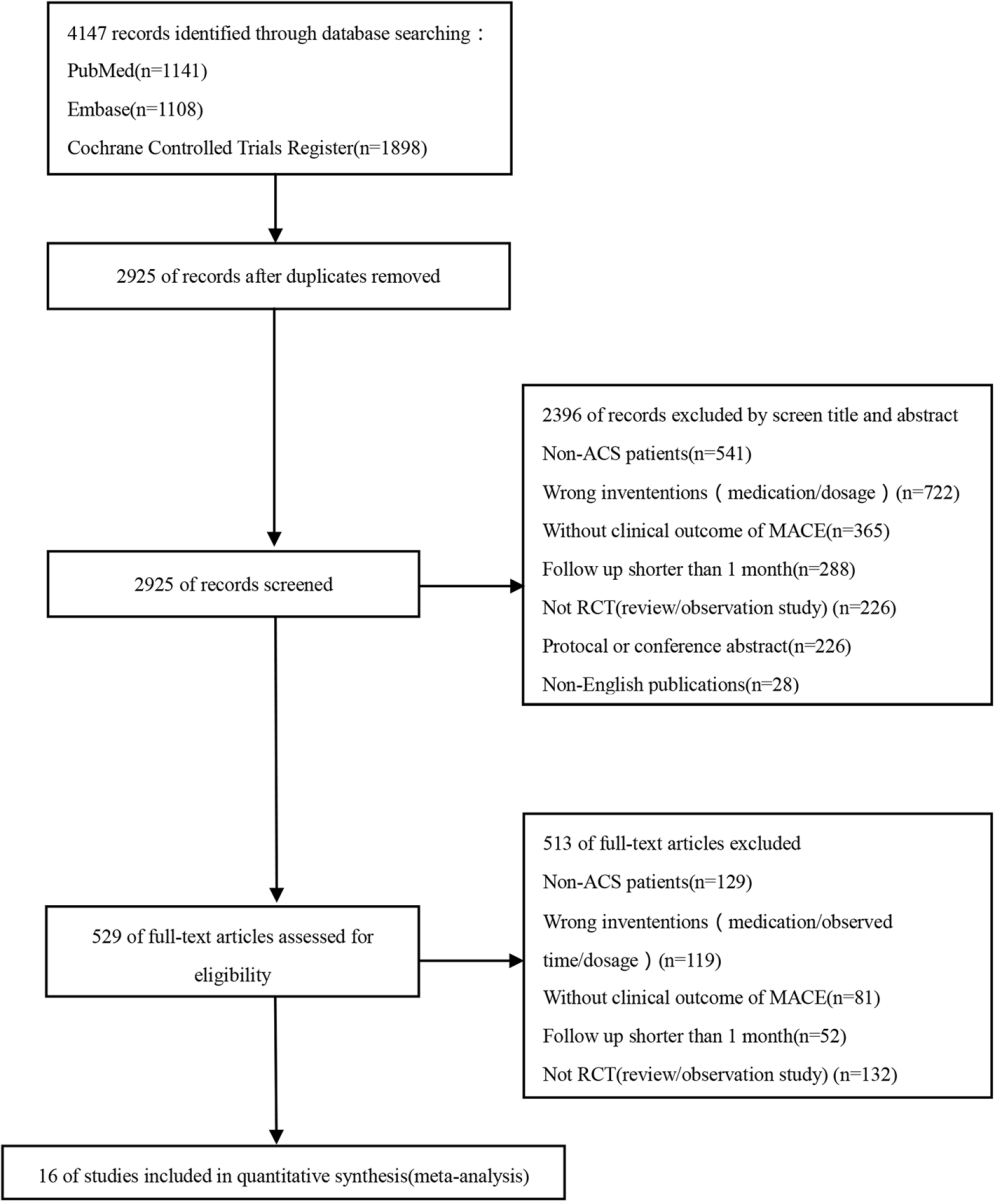
Flow diagram for the inclusion of identified trials. RCT, randomized controlled trial

A PRISMA-style flowchart was used to describe the study selection process. Of the 4147 potentially relevant articles, 16 eligible trials were finally identified (Fig. 1) [1, 6, 7, 16–28]. Among the 16 trials that included a total of 26,497 patients diagnosed with ACS, two were international multicenter studies; seven were multicenter trials performed in the Netherlands, UK, China, Korea, and Italy, respectively, and seven were single-center trials carried out in India, Egypt, and China (Table 1).

**Table 1:** Characteristics of 16 Trials Meeting Criteria for Inclusion in the Meta-analysis

Study	Diagnoses	Country	Age,	High-intensity statin	Standard statin	Follow-up Time
Armitage et al., 2010 [18]	History of MI	UK Multi-center trial	64.2 ± 8.9	Simvastatin 80 mg	Simvastatin 20 mg	2, 12, 24, and 36 months
Cannon et al., 2004 [1]	ACS	International Multi-center trial	58	Atorvastatin 80 mg	Pravastatin 40 mg	1, 3, 4, and 24 months
Colivicchi et al., 2010 [26]	Non-STEMI	Italy Multi-center trial	75.2 ± 9.9/73.9 ± 9.4	Atorvastatin 80 mg	Atorvastatin 20 mg	12 months
Colivicchi et al., 2002 [27]	UA or Non Q Wave MI	Italy Multi-center trial	69/68	Atorvastatin 80 mg	Usual care	1, 4, and 12 months
De Lemos et al., 2004 [28]	ACS	International Multi-center trial	61/61	Simvastatin 40 mg 1 month follow by 80 mg	Placebo 4 months follow by simvastatin 20 mg	24 months
Im et al., 2018 [25]	ACS	Korea Multi-center trial	64 ± 12/64 ± 12	Atorvastatin 40 mg	Pravastatin 20 mg	12 months
Guo et al., 2017 [24]	ACS after stent implantation	Chinese Single-center trial	57.8 ± 6.4/62.3 ± 3.7	Rosuvastatin 20 mg	Rosuvastatin 10 mg	3 months
Liu et al., 2019 [6]	Acute STEMI undergoing emergency PCI	Chinese Single-center trial	58.4/60.5	Atorvastatin 40 mg	Atorvastatin 20 mg	12 months
Liu et al., 2016 [21]	ACS requiring PCI	Chinese Single-center trial	61.8 ± 10.1/62.5 ± 11.2	Atorvastatin 80 mg	Atorvastatin 20 mg	1 and 12 months
Liu et al., 2016 [20]	ACS with DM underwent primary or Early PCI	Chinese Single-center trial	61.6 ± 8.7/62.1 ± 10.2	Atorvastatin 40 mg	Atorvastatin 20 mg	1 and 12 months
Priti et al., 2017 [19]	Acute STEMI	India Single-center trial	56.64/57.35	Atorvastatin 80 mg	Atorvastatin 10 mg	1 month
Shehata et al., 2015 [17]	NSTE-ACS	Egypt Single-center trial	58 ± 9/57 ± 8	Atorvastatin 80 mg	Atorvastatin 20 mg	6 months
Shehata et al., 2017 [16]	NSTE-ACS	Egypt Single-center trial	56 ± 9/55 ± 11	Atorvastatin 80 mg	Atorvastatin 20 mg	3 months
Pedersen et al., 2010 [23]	Had a first acute MI < 2 months before randomization	Dutch Multi-center trial	60.6 ± 9.7/59.8 ± 9.6	Atorvastatin 80 mg	Simvastatin 20 to 40 mg	over 5 years
Zhao et al., 2014 [7]	ACS	Chinese Multi-center trial	60.8/60.4	Atorvastatin 20 or 40 mg	Atorvastatin 10 mg	24 months
Zheng et al., 2015 [22]	NSTEMI	Chinese Multi-center trial	59.47 ± 8.6/59.70 ± 8.4	Atorvastatin 40 mg	Atorvastatin ≤20 mg	1 month

### Study characteristics

The mean sample size was 1656 (range, 81–11,945). High-intensity statin regimens comprised atorvastatin at 80 mg (8 studies), atorvastatin at 40 mg (5 studies), rosuvastatin at 20 mg (1 study), and simvastatin at 80 mg (2 studies). Mean patient age ranged between 56.5 and 75.2 years. Follow-up was performed for 12 months, on average (range, 1–60 months).

### Risk of methodological bias

**Fig. 2:**
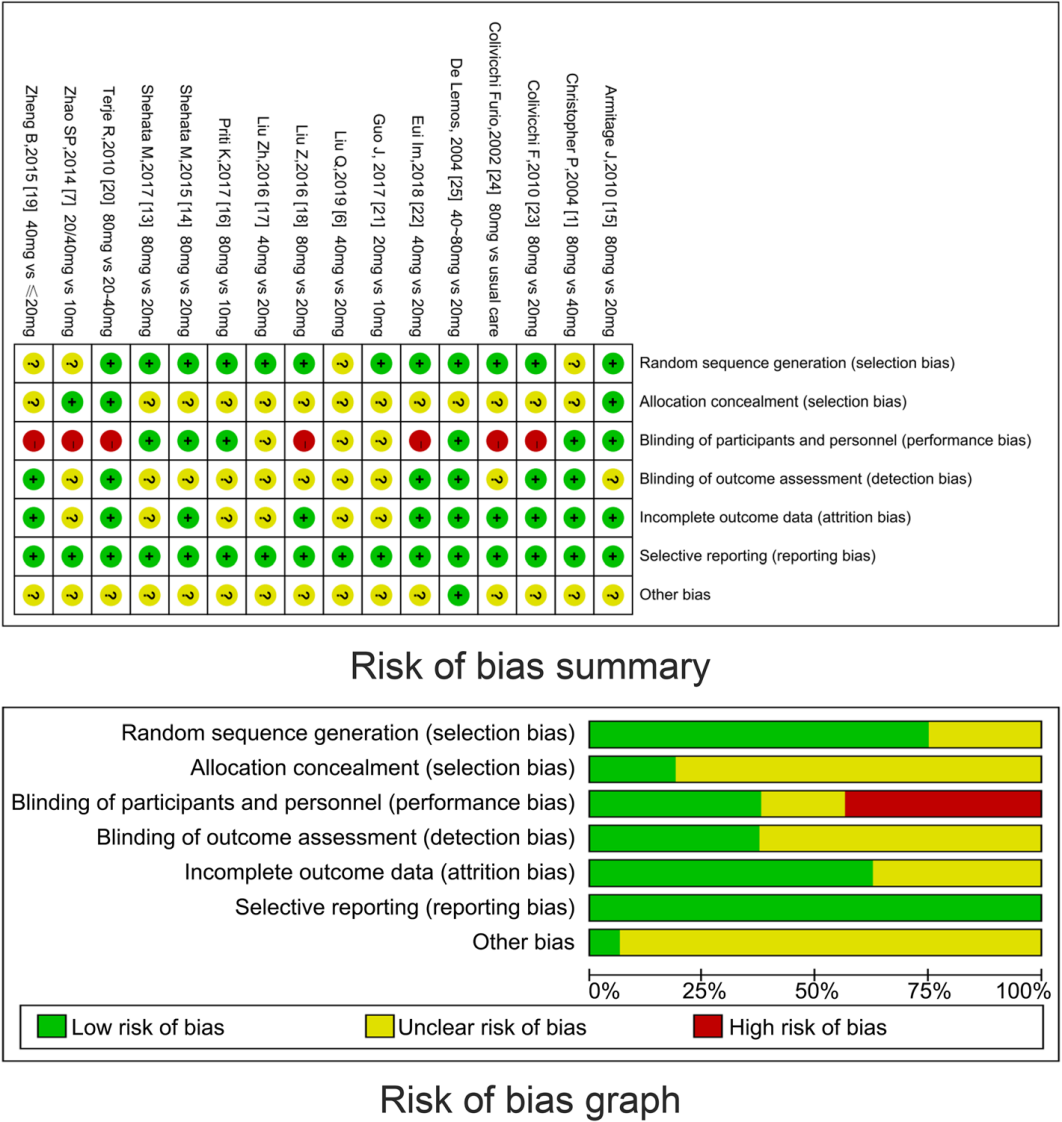
The risk of methodological bias. **a** Funnel plot analysis evaluating the effects of high-intensity statin treatment vs. standard statin administration on MACE. **b** Risk of methodological bias graph presenting the authors’ judgment about each risk of bias item shown as a percentage across all the included studies. **c** Evaluation grid of all included studies

Of the 16 trials, 12 reported a random sequence generation [1, 16–21, 23–28]. Three trials reported allocation concealment [7, 18, 23]. Six of the trials reported blinding of participants [1, 16–19, 28], and six reported blinding of outcome assessment [1, 22, 23, 25, 26, 28]. None of the trials reported bias for MACE. In summary, 16 trials showed at least two domains determined as an unclear risk of bias, seven showed blinding of participants determined as a high risk of bias. All the trials showed domain of selective reporting determined as low risk of bias (Fig. 2).

### Primary outcome

**Fig. 3:**
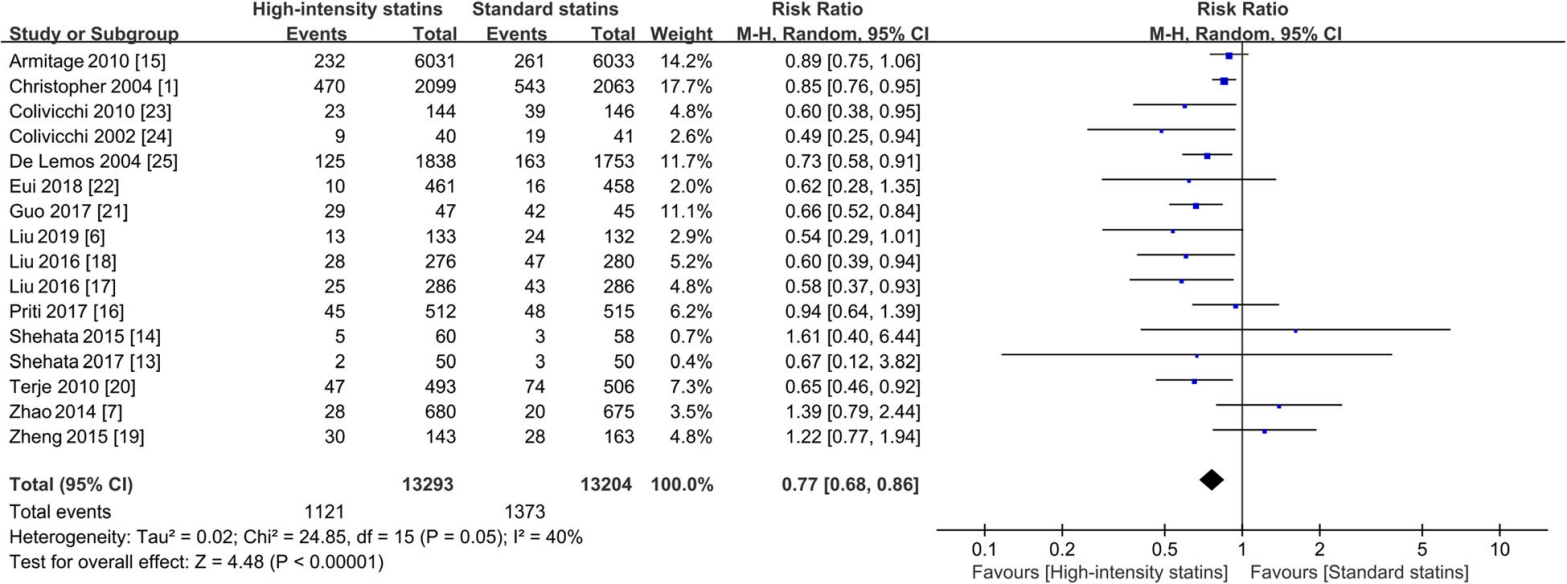
Forest plot of MACE. RR, risk ratio; M-H, Mantel-Haenszel method, MACE, major adverse cardiovascular events

A total of 16 studies reported 1121 (8.43%) incidences of MACE in 13,293 individuals of the high-intensity statin group vs. 1373 (10.40%) in 13,204 participants of the standard statin group. Compared with standard statin therapy, high-intensity statin administration reduced the risk of MACE (RR = 0.77; 95%CI, 0.68–0.86; *P* < 0.00001; prediction interval, 0.555–1.069), with heterogeneity among the 16 trials (I^2^ = 40%, *P* = 0.05), as shown in Fig. 3. Evaluations with the fixed-effects model yielded results that were highly consistent with the main raw finding above (RR = 0.81; 95%CI, 0.75–0.87; *P* < 0.00001) (Supplementary Figure 1). Simvastatin at 80 mg, which on average, lowers low-density lipoprotein cholesterol (LDL-C) by about 50%, is considered a high-intensity statin regimen, but not recommended by the Food and Drug Administration (FDA) due to increased risk of myopathy. A post hoc sensitivity analysis was carried out by excluding the published data of the two trials using simvastatin 80 mg. The pooled results from the remaining 14 trials were highly consistent with the main finding above (RR = 0.80; 95%CI, 0.74–0.87; I^2^ = 41%). Similarly, 11 studies reported HR of MACE, further analysis demonstrated that high-intensity statin treatment significantly reduced the risk of MACE in ACS patients (HR = 0.79; 95%CI, 0.75–0.83; *P* < 0.00001, Supplementary Figure 2).

### Subgroup analysis

**Fig. 4:**
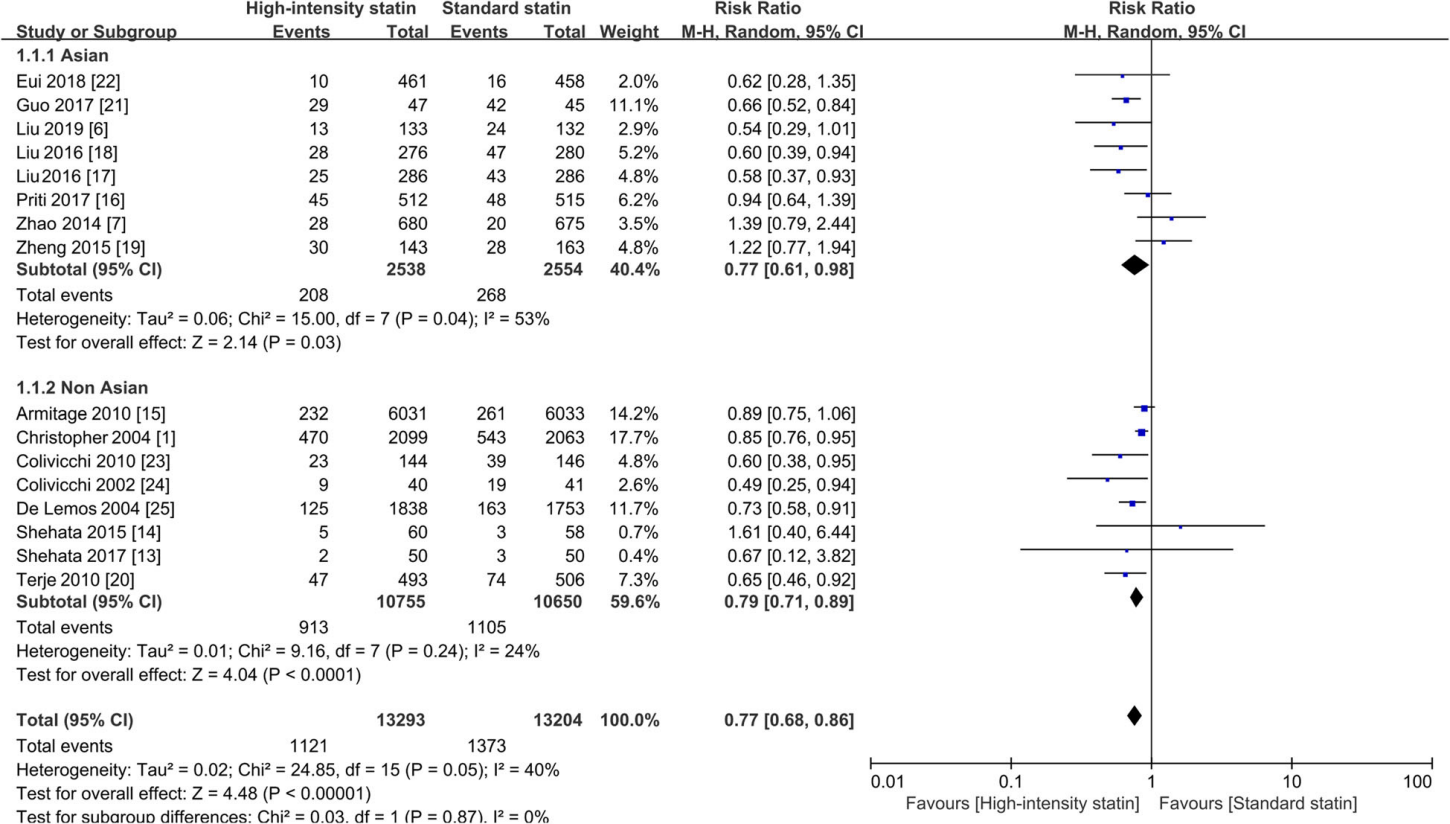
Forest plot of MACE by patient race. RR, risk ratio; M-H, Mantel-Haenszel method, MACE, major adverse cardiovascular events

A subgroup analysis was performed based on Asian and Non-Asian ethnicity. In the subgroup analysis of 5092 Asian patients, a significantly reduced incidence of MACE was observed after high-intensity treatment vs. standard statin therapy (8.20% vs. 10.49%), with a 23% decrease in the risk of MACE (RR = 0.77; 95%CI, 0.61–0.98; *P* = 0.03). In the 21,405 non-Asian participants, 913 (8.50%) out of 10,755 individuals reported MACE in the high-intensity statin group vs. 1105/10,650 (10.40%) in standard statin users, with a 21% reduction in the risk of MACE (RR = 0.79; 95%CI, 0.71–0.89; *P* < 0.0001). These results indicated that high-intensity statin therapy could yield more clinical benefits regarding MACE compared with a standard statin in both Asian and non-Asian patients, as shown in Fig. 4 (or in Supplementary Figure 3, using Peto ORs). A meta-regression showed no causal relationship between ethnicity and MACE (*P* > |t| = 0.963; exp. = 1.01, 95%CI: 0.75–1.35).

**Fig. 5:**
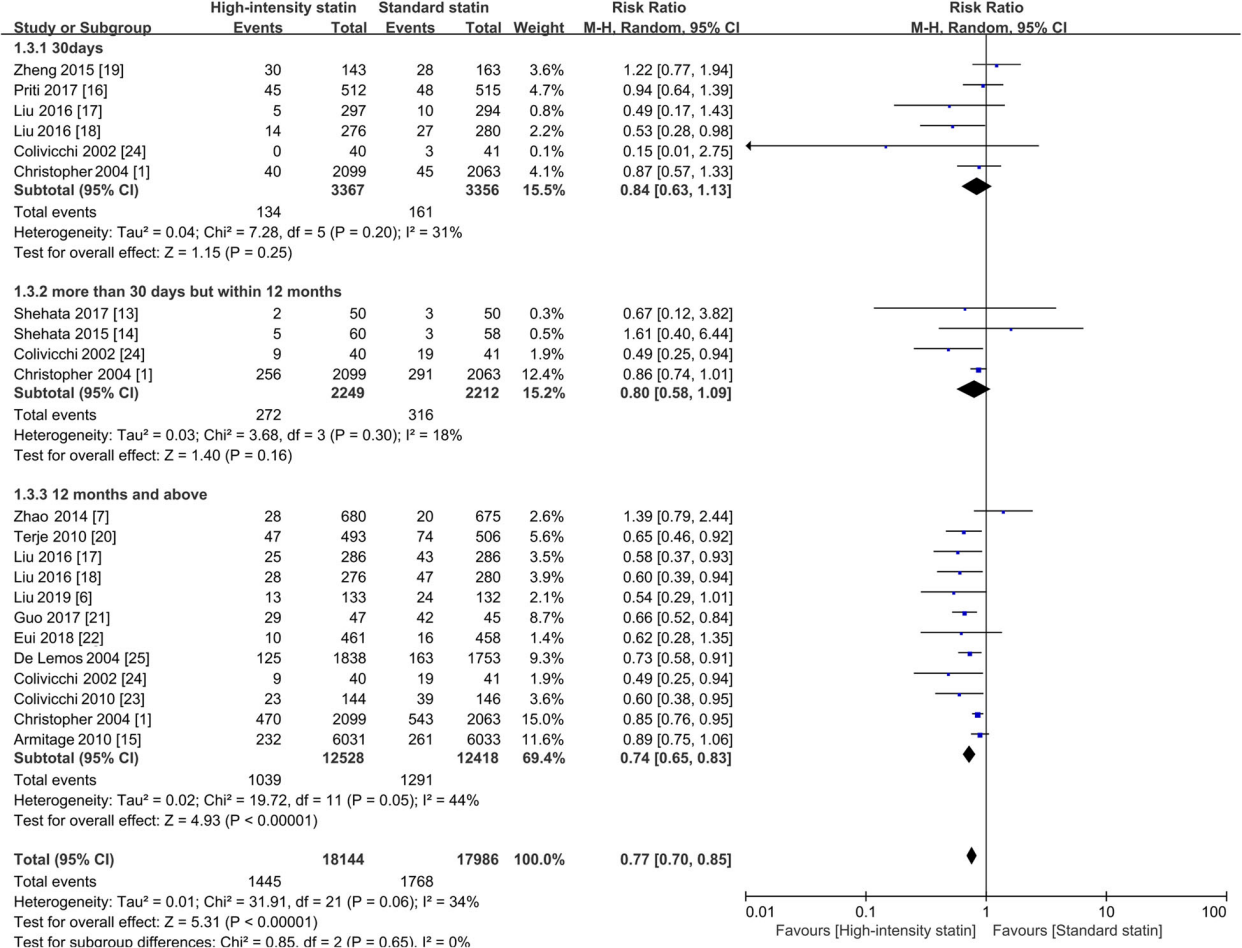
Forest plot of MACE by the duration of treatment. RR, risk ratio; M-H, Mantel-Haenszel method, MACE, major adverse cardiovascular events

There was no reduction in the risk of MACE in the first month following high-intensity statin treatment (RR = 0.84; 95%CI, 0.63–1.13; *P* = 0.25). Between 30 days and 12 months, there was a decreasing trend in the risk of MACE (RR = 0.80; 95%CI, 0.58–1.09; *P* = 0.03, I^2^ = 18%). A benefit conferred by high-intensity statin occurred after > 12 months of treatment (RR = 0.74; 95%CI, 0.65–0.83) with heterogeneity (I^2^ = 44%, *P* = 0.05), as shown in Fig. 5 (or in Supplementary Figure 4, using Peto ORs).

### Secondary outcomes

**Fig. 6:**
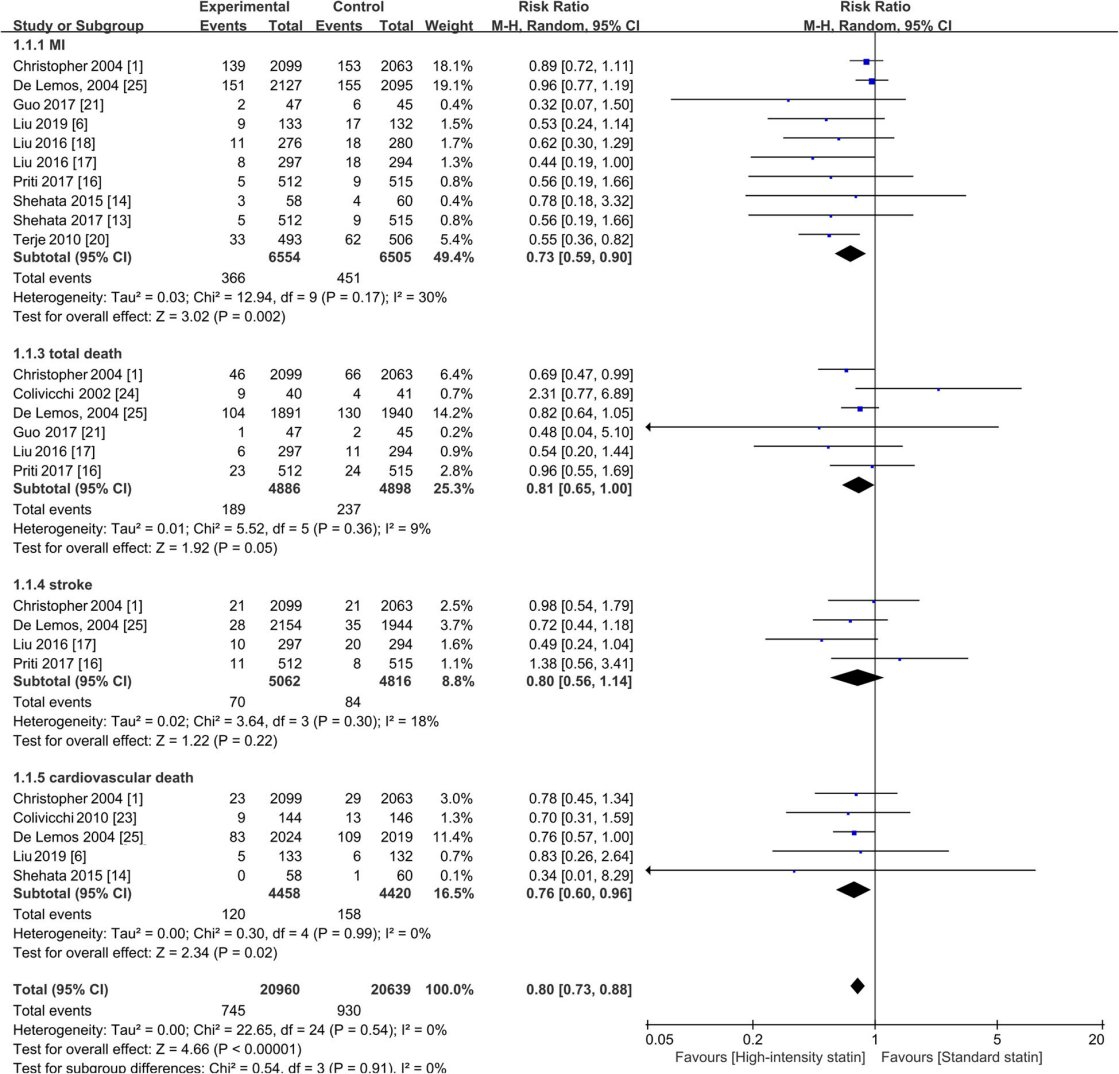
Forest plot of secondary outcomes. RR, risk ratio; M-H, Mantel-Haenszel method

High-intensity statin treatment also reduced the risks of recurrent myocardial infarction (RR = 0.73; 95%CI, 0.59–0.90; I^2^ = 30%; prediction interval, 0.460–1.158) and cardiovascular death (RR = 0.76; 95%CI, 0.60–0.96; I^2^ = 0%; prediction interval, 0.545–1.060), but high-intensity statin treatment did not decrease total death (RR = 0.81; 95%CI, 0.65–1.00; I^2^ = 9%; prediction interval, 0.552–1.188) and stroke risk (RR = 0.80; 95%CI, 0.56–1.14; I^2^ = 18%; prediction interval, 0.385–1.663) as shown in Fig. 6 (or in Supplementary Figure 5, using Peto ORs). A meta-regression showed no causal relationship between type of acute coronary syndrome and MACE (*P* > |t| = 0.841; exp. = 1.03, 95%CI: 0.76–1.40).

### Lipid-lowering effects

Nine RCTs, with a total of 9785 patients, reported the LDL-C parameter. The mean LDL-C reduction was 0.83 mmol/l (23.1% decline) in the standard statin therapy group, while there was a more pronounced reduction in participants administered high-intensity statin (1.37 mmol/l, 44.7% decline). The discrepancy in LDL-C reduction between the high-intensity and standard statin groups was calculated (MD = 0.54; 95%CI, 0.23–0.84). Following the subgroup pooled analysis of the nine trials that reported the LDL-C index, high-intensity statin treatment also decreased the risk of MACE compared with standard statin administration in ACS cases (RR = 0.78; 95%CI, 0.70–0.86; I^2^ = 31%). A meta-regression showed no causal relationship between LDL-C and MACE (*P* > |t| = 0.582; exp. = 1.16, 95%CI: 0.63–2.13). The differential effects of different statin regimens should be explored by subgroup analysis and presented in Supplementary Figure 6. A meta-regression showed no causal relationship between different regimens and MACE (*P* > |t| = 0.961; exp. = 1.00, 95%CI: 0.92–1.09).

### Adverse events

Among the 16 trials, five reported that there were 164 individuals (4.99%) administered high-intensity statin therapy vs. 97 (2.98%) administered standard statin therapy who developed myopathy or myalgia. Rhabdomyolysis was observed in one patient in the IDEAL subgroup study who received standard statin treatment. Meanwhile, five studies reported that 96 individuals (1.84%) in the high-intensity statin group had elevated serum aminotransferase and/or liver aminotransferase amounts (3 × upper limit of normal) vs. 42 individuals (0.84%) in the standard statin group.

### Publication bias

**Fig. 7:**
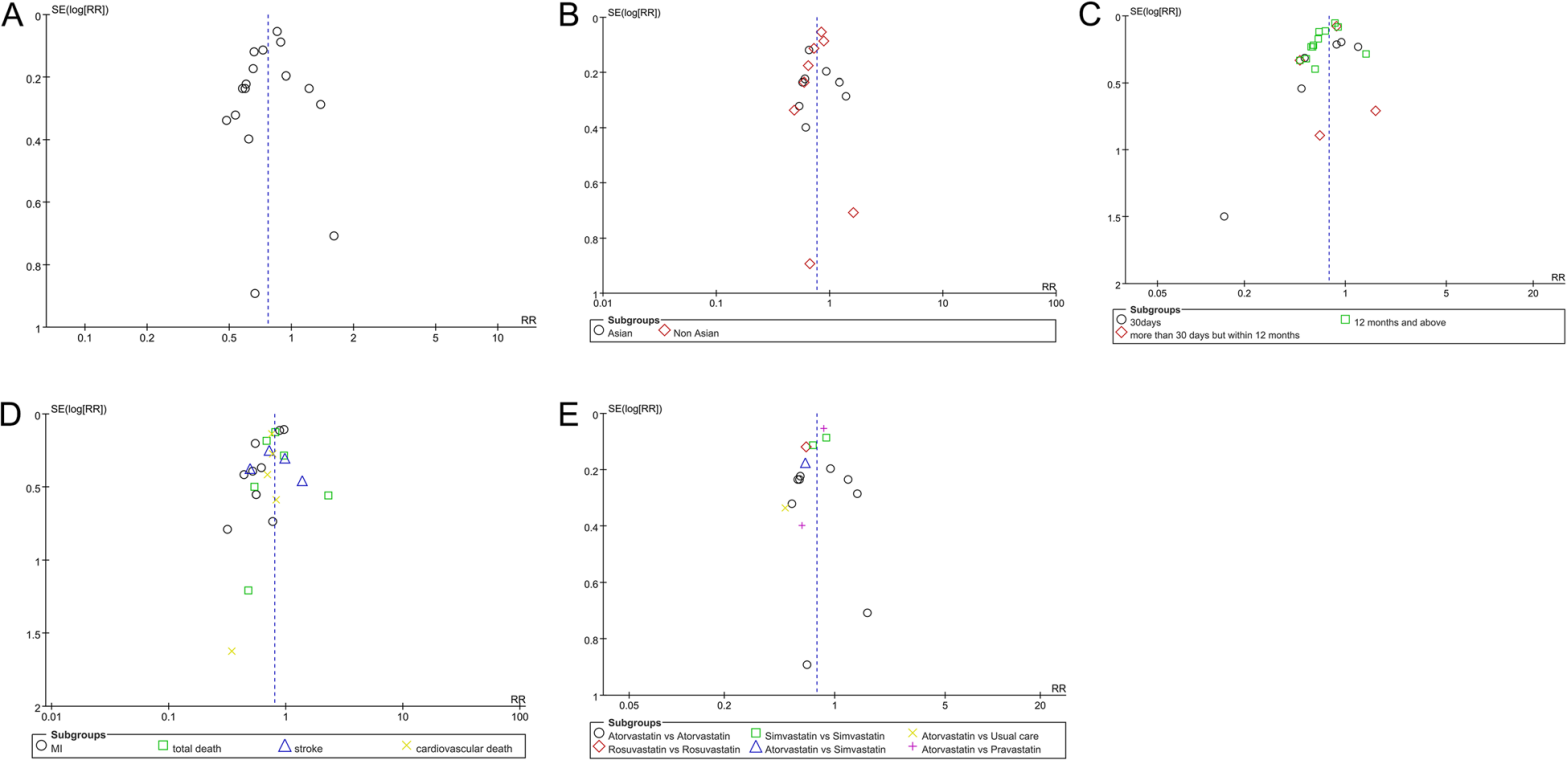
The publication bias analysis. **a** MACE. **b** Ethnicity. **c** Different follow-up. **d** Secondary outcomes. **e** Different statin regimens. SE, standard error; RR, relative risk

According to the funnel plots and Egger/Begg’s tests (Fig. 7), no publication bias was shown in these trials for MACE (*P* = 0.324), total death (*P* = 0.759), stroke (*P* = 0.703), cardiovascular death (*P* = 0.326), and the results were objectively reported. The Egger test showed the existence of bias for MI (*P* = 0.008).

### Quality of evidence

Seven outcomes were included in the meta-analysis, and all seven outcomes except pruritus were important results. GRADE Working Group levels of evidence were moderate for MACE and low for myocardial infarction, total death, stroke, and cardiovascular death (Table 2).

**Table 2:** GRADE evidence profile. High-intensity statin compared with a standard statin for acute coronary syndrome patients. Patient or population: 26,497 with acute coronary syndrome. Settings: worldwide. Intervention: High-intensity statin. Comparison: Standard statin

Outcomes	Illustrative comparative risks^a^ (95% CI)		Relative effect	Prediction interval	Estimated probability for RR ≥ 1	No of Participants (studies)	Risk of bias	Inconsistency	Indirectness	Impression	Reporting bias	Comments	Quality of the evidence (GRADE)
	Assumed risk	Corresponding risk	(95% CI)										
	Standard statin	High-intensity statin											
MACE	Low risk population		0.77	(0.555, 1.069)	5.50%	26,497	All the studies were RCTS, but some studies were open-labled.	The heterogeneity is relatively low I^c^ = 40%, Tau^c^ = 0.02	All the studies compared statins with each other, not indirectly	Not serious^b^	No		⊕ ⊕ ⊕⊝
	104 per 1000	84 per 1000	(0.68 to 0.86)			(16)							moderate
		(22 to 617)											
MI	Medium risk population		0.73	(0.460, 1.158)	7.90%	13,059	All the studies were RCTs. No assessment of publication bias made for this outcome. But some studies were open-labled	The heterogeneity is relatively low I^c^ = 30%, Tau^c^ = 0.03	All the studies compared statins with each other, not indirectly	Not serious^b^	All the included studies had positive results, which may have publication bias	Downgraded by 1 level.	⊕ ⊕ ⊝ ⊝
	69 per 1000	56 per 1000	(0.59 to 0.90)			(10)						See footnotes.	low
		(10 to 71)											
Total death	Medium risk population		0.81	(0.552, 1.188)	10.80%	9784	All the studies were RCTS, but some studies were open-labled	The heterogeneity is relatively low I^c^ = 9%, Tau^c^ = 0.01	All the studies compared statins with each other, not indirectly	Not serious^c^	No	Downgraded by 1 level.	⊕ ⊕ ⊝ ⊝
	48 per 1000	39 per 1000	(0.65 to 1.00)			(6)						See footnotes.	low
		(21 to 225)											
Stroke	Medium risk population		0.8	(0.385, 1.663)	20.20%	9878	All the studies were RCTS. But some studies were open-labled	The heterogeneity is relatively low I^c^ = 18%, Tau^c^ = 0.02	All the studies compared statins with each other, not indirectly	Not serious^c^	No	Downgraded by 1 level.	⊕ ⊕ ⊝ ⊝
	17 per 1000	14 per 1000	(0.56 to 1.14)			(4)				Only 4 studies with event data. Downgraded for imprecision.		See footnotes.	low
		(10 to 34)											
Cardiovascular death	Medium risk population		0.76	(0.545, 1.060)	4.20%	8878	All the studies were RCTS. But some studies were open-labled	The heterogeneity is relatively low I^c^ = 0%, Tau^c^ = 0.00	All the studies compared statins with each other, not indirectly	Not serious^b^	All the included studies had positive results, which may have publication bias	Downgraded by 1 level.	⊕ ⊕ ⊝ ⊝
			(0.69 to 0.83)			-(5)				Only 5 studies with event data. Downgraded for imprecision.			low

^a^The basis for the assumed risk (e.g., the median control group risk across studies) is provided in footnotes. The corresponding risk (and its 95% confidence interval) is based on the assumed risk in the comparison group and the relative effect of the intervention (and its 95% CI)

^b^ *P* < 0.05 for CI, but no significant effect considering the PI;

^c^ no significant effect basing on CI and PI;

*CI* Confidence interval, *PI* Prediction interval, *RR* Risk Ratio;

GRADE Working Group grades of evidenceHigh quality: Further research is very unlikely to change confidence in the effect estimate.Moderate quality: Further research is likely to have an important impact on confidence in the effect estimate and may change the estimate.Low quality: Further research is very likely to have an important impact on confidence in the effect estimate and is likely to change the estimate.Very low quality: The estimate is very uncertain

## Discussion

The present meta-analysis first assessed the effects of high-intensity statin vs. standard statin on MACE in ACS. The resulting findings might have certain implications for clinical decision-making. First, 16 RCTs evaluating 26,497 patients with ACS were included and a superiority of high-intensity statin administration over standard statin therapy towards MACE was observed in the raw analysis, but a random-effect was used and the association was not significant according to the prediction intervals. Second, based on the time-course of statin treatment in subgroup analysis, the benefits of high-intensity statin therapy were visible within 12 months compared with standard statin administration. Third, similar to non-Asian ACS patients, Asian participants could benefit more from high-intensity statin therapy compared to standard statin treatment.

This updated meta-analysis of 16 RCTs showed in the raw analysis that high-intensity statin therapy yields significant benefits towards MACE. In their observational studies, Kim and Dennis also reported that high-intensity statin-treated individuals have markedly reduced the incidence of MACEs in comparison with the non-high-intensity statin group [25, 29]. A previous meta-analysis of RCTs that included a total of 39,612 patients with coronary heart disease also showed that high-intensity statin therapy further decreases MACE incidence [30]. Yet, only two trials assessing 8659 ACS patients did not include the pooled results on MACE. Generally speaking (i.e., when using the raw analysis and the subgroup analyses by race), the present meta-analysis agrees with a previous one [5], but when considering the prediction interval in all patients, the association becomes non-significant and agree with two other meta-analyses [6, 7]. Hence, the controversy remains.

### Statin intensity-based strategy vs. LDL-C target-based strategy

Two major guidelines by the American College of Cardiology/American Heart Association (ACC/AHA) and the European Society of Cardiology (ESC) co-presented with European Atherosclerosis Society (EAS) recommend different lipid-lowering strategies for secondary prevention in ACS cases [31]. The ACC/AHA guidelines advocate for evidence-based intensity statin treatment without specific cholesterol targets, whereas the ESC/EAS guidelines focus on decreasing LDL-C to specific treatment targets. Such a difference between these two guidelines has led to confusion in the clinical setting. According to the “statin hypothesis”, statins ameliorate patient outcome via biologic effects no associated with their ability to decrease cholesterol levels, notably their anti-inflammatory features [32]. However, the large IMPROVE-IT trial suggested that the primary goal of LDL cholesterol-lowering as a strategy to prevent coronary heart disease [33]. This confirms the “LDL hypothesis” describing the relation between LDL-C and cardiovascular events [34]. In addition, genetic analyses have revealed causality relation between circulating LDL-C and cardiovascular, corroborating molecular and epidemiological findings; therefore, the “LDL-hypothesis” was suggested to be substituted by “LDL causality” [34]. Nevertheless, there are limited data directly comparing statin intensity-based and LDL-C target-based strategies. It was shown that pharmacologically inhibiting cholesterol absorption (using ezetimibe) and PCSK9 activity (using evolocumab or alirocumab) constitute efficient options for modulating LDL-C metabolism in patients administered statin; indeed, combining statin and non-statin agents alleviates coronary atherosclerosis, with overt cardiovascular benefits in moderate-to-high cardiovascular risk individuals [35–37]. Interestingly, a study found no association of LDL-C with cognitive function alterations [38], although depression was not assessed in this cohort [39]. In the latter study, trials with ezetimibe or evolucumab and alirocumab were not included. Subgroup analysis in this study revealed more pronounced reductions of LDL-C and MACE risk in trials applying high-intensity statin treatment compared with the standard statin group. Interestingly, it has been shown that a tailored treatment strategy with high-dose statin could better prevent coronary artery disease events than LDL-C-based target approaches [40].

Despite convincing data published by international multicenter trials, the benefits of high-intensity statin in the Asian population remain unclear. It seems that patients in Asia may have better statin responsiveness compared with North Americans and Europeans, and standard statin treatment might be sufficient in Asian patients. However, high-intensity statin therapy is not widely implemented in Asians [41–43]. Recently, even in Asian patients living in Europe, high-intensity statin prescriptions at discharge are 6.3% lower than those prescribed to Caucasians [44]. Interestingly, the magnitude of relative risk reduction of MACE in Asian patients administered high-intensity statin treatment was comparable to that of non-Asian subjects, as shown above. This indicates that like non-Asian patients, Asian ACS cases could benefit more from high-intensity statin treatment compared with standard statin administration.

The benefits of high-intensity statin treatment took more than 1 month to occur in the present study. The JAPAN-ACS study demonstrated that statin administration significantly inhibits coronary atherosclerosis only following 8 to 12 months of treatment [45]. A previous meta-analysis also reported that high-intensity statin treatment does not result in plaque regression during the first 3 months in ACS patients; however, plaque regression occurred after 6–12 months and persisted for more than 12 months [46].

### Adverse events

High-intensity statins are commonly avoided for feared adverse event induction. This meta-analysis demonstrated that high-intensity and standard statin regimens had acceptable rates of adverse events. In addition, serious adverse events were rare.

### Implications for practice

The present results confirm the effectiveness of high-intensity statin administration in ACS. Accordingly, pooled results might provide the impetus for the guidelines committee, who should upgrade the evidence level B of high-intensity statin recommendation in the 2013 ACCF/AHA Guideline for the Management of ST-Elevation Myocardial Infarction [3]. High-intensity statin therapy should be recommended by the ACS management guidelines, especially in the Asian context. However, close attention should be paid to long-term adherence to statins in recently detected ACS cases. Nevertheless, the outcomes were analyzed using prediction intervals [14], which are prediction intervals for random-effect models indicating the probable true treatment effect in future settings, and to perform better power calculations. Nevertheless, the predicted interval would be precise only under the assumption that the τ^2^ value is precise. Nevertheless, in the present study, the prediction intervals only include the null hypothesis instead than the opposite results, which is less worrying [14].

### Study strengths and limitations

There are numerus strengths, first, this updated Meta-analysis enrollment the biggest population, and subgroup analysis was performed as well as meta-regression. However, the present study had multiple limitations. First, the present meta-analysis was performed without a prior registry. Nevertheless, the primary endpoint is not based on post hoc analysis. Second, the outcomes described in the assessed trials were employed, and whether distinct baseline features could alter these findings remains unknown. Third, multiple baseline features, including older age, hypertension, diabetes, and kidney function impairment, which may influence patient prognosis, were not included in this analysis, potentially causing mixed bias. Fourth, some analyses suffered from heterogeneity, but GRADE analysis nevertheless showed low to moderate quality of evidence for the primary outcomes. Fifth, this study presents a publication bias for reducing the risk of MI. Therefore, the interpretation should be cautious for MI. Sixth, a cost-benefit assessment that could help evaluate potential savings associated with the abovementioned benefits of this intensive secondary prevention approach was not performed. Finally, this meta-analysis was nor registered and is therefore not PRISMA-compliant.

### Perspectives and clinical relevance

Based on these shortcomings, multidisciplinary studies should be performed to comprehensively evaluate the benefits of high-intensity statin treatment, to provide a broad application of this beneficial therapeutic approach. Nevertheless, even if improvements can be achieved in the amount and quality of data, GRADE analysis showed that the quality of the MACE data was moderate, indicating that additional studies are unlikely to substantially change the effect estimate. Nevertheless, studies could be performed to identify which categories of patients might benefit the most from high-intensity statin treatment. The application of this regimen would improve the quality of life of ACS patients and alleviate the burden on the families and the society at large. In a future study with similar setting, the estimated probability that a true RR ≥ 1 for high-intensity statin therapy is equal to 5.5, 7.9, 10.8, 20.2, 4.2% for MACE, MI, total death, stroke, cardiovascular death respectively.

## Conclusion

The current findings indicated that high-intensity statin treatment might reduce MACE in ACS in comparison with standard statin therapy, but the prediction interval suggests that it might not be the case in all patients. In addition, serious adverse events associated with high-intensity statin administration were rare.

## Supplementary information

**Additional file 1: Figure S1.** Forest plot of MACE, a) M-H for the fixed-effect model; b) Peto method for the fixed-effect model. RR, risk ratio; M-H, Mantel-Haenszel method; MACE, major adverse cardiovascular events.

**Additional file 2: Figure S2.** Forest plot of MACE. HR, Hazard Ratio; M-H, Mantel-Haenszel method, MACE, major adverse cardiovascular events.

**Additional file 3: Figure S3.** Forest plot of MACE by patient race. RR, risk ratio; MACE, major adverse cardiovascular events.

**Additional file 4: Figure S4.** Forest plot of MACE by the duration of treatment. RR, risk ratio; MACE, major adverse cardiovascular events.

**Additional file 5: Figure S5.** Forest plot of the secondary outcomes. RR, risk ratio.

**Additional file 6: Figure S6.** Differential effects of different statin regimens in the subgroup analysis.

## Data Availability

The datasets used and/or analyzed during the current study are available from the corresponding author on reasonable request.

## Abbreviations

ACS: Acute Coronary Syndromes
MI: Myocardial infarction
MACE: Major adverse cardiovascular events
MeSH: Medical subject headings
CI: Confidence intervals
LDL-C: Low-density lipoprotein cholesterol
RR: Relative risk
FDA: Food and Drug administration
ACC/AHA: American College of Cardiology/American Heart Association
ESC: European Society of Cardiology
EAS: European Atherosclerosis Society

## References

[1] Cannon CP, Braunwald E, McCabe CH, Rader DJ, Rouleau JL, Belder R (2004). Intensive versus moderate lipid lowering with statins after acute coronary syndromes. N Engl J Med.

[2] Schwartz GG, Olsson AG, Ezekowitz MD, Ganz P, Oliver MF, Waters D (2001). Effects of atorvastatin on early recurrent ischemic events in acute coronary syndromes: the MIRACL study: a randomized controlled trial. Jama..

[3] O'Gara PT, Kushner FG, Ascheim DD, Casey DE, Chung MK, de Lemos JA (2013). 2013 ACCF/AHA guideline for the management of ST-elevation myocardial infarction: a report of the American College of Cardiology Foundation/American Heart Association task force on practice guidelines. Circulation..

[4] Vale N, Nordmann AJ, Schwartz GG, et al. Statins for acute coronary syndrome. Cochrane Database Syst Rev. 2014;(9):CD006870. 10.1002/14651858.CD006870.pub3 PMC11126893 25178118

[5] Cannon CP, Steinberg BA, Murphy SA, Mega JL, Braunwald E (2006). Meta-analysis of cardiovascular outcomes trials comparing intensive versus moderate statin therapy. J Am Coll Cardiol.

[6] Liu Q, Wang Y, Cheng X (2019). The functional effect of atorvastatin dose-dependent via inflammation factors on acute ST segment elevation myocardial infarction after emergency percutaneous coronary intervention. J Cardiovasc Med (Hagerstown).

[7] Zhao SP, Yu BL, Peng DQ, Huo Y (2014). The effect of moderate-dose versus double-dose statins on patients with acute coronary syndrome in China: results of the CHILLAS trial. Atherosclerosis..

[8] Stone NJ, Robinson JG, Lichtenstein AH, Bairey Merz CN, Blum CB, Eckel RH (2014). 2013 ACC/AHA guideline on the treatment of blood cholesterol to reduce atherosclerotic cardiovascular risk in adults: a report of the American College of Cardiology/American Heart Association task force on practice guidelines. Circulation..

[9] Arnett DK, Blumenthal RS, Albert MA, Buroker AB, Goldberger ZD, Hahn EJ (2019). 2019 ACC/AHA guideline on the primary prevention of cardiovascular disease. Circulation.

[10] Zhou JG, Tian X, Wang X, Tian JH, Wang Y, Wang F (2015). Treatment on advanced NSCLC: platinum-based chemotherapy plus erlotinib or platinum-based chemotherapy alone? A systematic review and meta-analysis of randomised controlled trials. Med Oncol.

[11] Higgins JP, Altman DG, Gotzsche PC, Juni P, Moher D, Oxman AD (2011). The Cochrane Collaboration's tool for assessing risk of bias in randomised trials. BMJ..

[12] Morley LC, Tang T, Yasmin E, Norman RJ, Balen AH (2017). Insulin-sensitising drugs (metformin, rosiglitazone, pioglitazone, D-chiro-inositol) for women with polycystic ovary syndrome, oligo amenorrhoea and subfertility. Cochrane Database Syst Rev.

[13] Guyatt GH, Oxman AD, Kunz R, Vist GE, Falck-Ytter Y, Schünemann HJ (2008). What is “quality of evidence” and why is it important to clinicians?. Bmj..

[14] IntHout J, Ioannidis JP, Rovers MM, Goeman JJ (2016). Plea for routinely presenting prediction intervals in meta-analysis. BMJ Open.

[15] Hulten E, Jackson JL, Douglas K, George S, Villines TC (2006). The effect of early, intensive statin therapy on acute coronary syndrome: a meta-analysis of randomized controlled trials. Arch Intern Med.

[16] Shehata M, Samir A, Dardiri M (2017). Prognostic impact of intensive statin therapy on N-terminal pro-BNP level in non-ST-segment elevation acute myocardial infarction patients. J Interv Cardiol.

[17] Shehata M, Fayez G, Nassar A (2015). Intensive statin therapy in NSTE-ACS patients undergoing PCI: clinical and biochemical effects. Tex Heart Inst J.

[18] Armitage J, Bowman L, Wallendszus K, Bulbulia R, Rahimi K, Haynes R (2010). Intensive lowering of LDL cholesterol with 80 mg versus 20 mg simvastatin daily in 12,064 survivors of myocardial infarction: a double-blind randomised trial. Lancet..

[19] Priti K, Agrawal A, Ranwa BL (2017). High versus low dose statin therapy in Indian patients with acute ST-segment elevation myocardial infarction undergoing thrombolysis. Indian Heart J.

[20] Liu Z, Xu Y, Hao H, Yin C, Xu J, Li J (2016). Efficacy of high intensity atorvastatin versus moderate intensity atorvastatin for acute coronary syndrome patients with diabetes mellitus. Int J Cardiol.

[21] Liu Z, Joerg H, Hao H, Xu J, Hu S, Li B (2016). Efficacy of high-intensity atorvastatin for Asian patients undergoing percutaneous coronary intervention. Ann Pharmacother.

[22] Zheng B, Jiang J, Liu H, Zhang J, Li H, Su X (2015). Efficacy and safety of serial atorvastatin load in Chinese patients undergoing elective percutaneous coronary intervention: results of the ISCAP (intensive statin therapy for Chinese patients with coronary artery disease undergoing percutaneous coronary intervention) randomized controlled trial. Eur Heart J Suppl.

[23] Pedersen TR, Cater NB, Faergeman O, Kastelein JJ, Olsson AG, Tikkanen MJ (2010). Comparison of atorvastatin 80 mg/day versus simvastatin 20 to 40 mg/day on frequency of cardiovascular events late (five years) after acute myocardial infarction (from the incremental decrease in end points through aggressive lipid lowering [IDEAL] trial). Am J Cardiol.

[24] Guo J, Zhang WZ, Zhao Q, Wo JS, Cai SL (2017). Study on the effect of different doses of rosuvastatin on ventricular remodeling in patients with acute coronary syndrome after emergency percutaneous coronary intervention. Eur Rev Med Pharmacol Sci.

[25] Im E, Cho YH, Suh Y, Cho DK, Her AY, Kim YH (2018). High-intensity statin treatments in clinically stable patients on aspirin Monotherapy 12 months after drug-eluting stent implantation: a randomized study. Revista Espanola de Cardiologia.

[26] Colivicchi F, Tubaro M, Mocini D, Genovesi Ebert A, Strano S, Melina G (2010). Full-dose atorvastatin versus conventional medical therapy after non-ST-elevation acute myocardial infarction in patients with advanced non-revascularisable coronary artery disease. Curr Med Res Opin.

[27] Colivicchi F, Guido V, Tubaro M, Ammirati F, Montefoschi N, Varveri A (2002). Effects of atorvastatin 80 mg daily early after onset of unstable angina pectoris or non-Q-wave myocardial infarction. Am J Cardiol.

[28] de Lemos JA, Blazing MA, Wiviott SD, Lewis EF, Fox KA, White HD (2004). Early intensive vs a delayed conservative simvastatin strategy in patients with acute coronary syndromes: phase Z of the a to Z trial. JAMA..

[29] Ko DT, Wijeysundera HC, Jackevicius CA, Yousef A, Wang J, Tu JV (2013). Diabetes mellitus and cardiovascular events in older patients with myocardial infarction prescribed intensive-dose and moderate-dose statins. Circ Cardiovasc Qual Outcomes.

[30] Baigent C, Blackwell L, Emberson J, Holland LE, Reith C, Bhala N (2010). Efficacy and safety of more intensive lowering of LDL cholesterol: a meta-analysis of data from 170,000 participants in 26 randomised trials. Lancet..

[31] Gencer B, Koskinas KC, Raber L, Karagiannis A, Nanchen D, Auer R (2017). Eligibility for PCSK9 inhibitors according to American College of Cardiology (ACC) and European Society of Cardiology/European atherosclerosis society (ESC/EAS) guidelines after acute coronary syndromes. J Am Heart Assoc.

[32] Joshi PH, Jacobson TA (2010). Therapeutic options to further lower C-reactive protein for patients on statin treatment. Curr Atheroscler Rep.

[33] Cannon CP, Blazing MA, Giugliano RP, McCagg A, White JA, Theroux P (2015). Ezetimibe added to statin therapy after acute coronary syndromes. N Engl J Med.

[34] DuBroff R, de Lorgeril M (2015). Cholesterol confusion and statin controversy. World J Cardiol.

[35] Gragnano F, Calabro P (2018). Role of dual lipid-lowering therapy in coronary atherosclerosis regression: evidence from recent studies. Atherosclerosis..

[36] Bove M, Fogacci F, Cicero AFG (2017). Pharmacokinetic drug evaluation of ezetimibe + simvastatin for the treatment of hypercholesterolemia. Expert Opin Drug Metab Toxicol.

[37] Strilchuk L, Tocci G, Fogacci F, Cicero AFG (2020). An overview of rosuvastatin/ezetimibe association for the treatment of hypercholesterolemia and mixed dyslipidemia. Expert Opin Pharmacother.

[38] Giugliano RP, Mach F, Zavitz K, Kurtz C, Im K, Kanevsky E (2017). Cognitive function in a randomized trial of Evolocumab. N Engl J Med.

[39] Giugliano RP, Sabatine MS, Ott BR (2017). Cognitive function in a randomized trial of Evolocumab. N Engl J Med.

[40] Hayward RA, Krumholz HM, Zulman DM, Timbie JW, Vijan S (2010). Optimizing statin treatment for primary prevention of coronary artery disease. Ann Intern Med.

[41] Khang AR, Song YS, Kim KM, Moon JH, Lim S, Park KS (2016). Comparison of different statin therapy to change low-density lipoprotein cholesterol and high-density lipoprotein cholesterol level in Korean patients with and without diabetes. J Clin Lipidol.

[42] Minami Y, Wang Z, Aguirre AD, Ong DS, Kim CJ, Uemura S (2017). Clinical predictors for lack of favorable vascular response to statin therapy in patients with coronary artery disease: a serial optical coherence tomography study. J Am Heart Assoc.

[43] Taguchi I, Iimuro S, Iwata H, Takashima H, Abe M, Amiya E (2018). High-dose versus low-dose Pitavastatin in Japanese patients with stable coronary artery disease (REAL-CAD): a randomized superiority trial. Circulation..

[44] Guedeney P, Baber U, Claessen B, Aquino M, Camaj A, Sorrentino S (2019). Temporal trends, determinants, and impact of high-intensity statin prescriptions after percutaneous coronary intervention: results from a large single-center prospective registry. Am Heart J.

[45] Hiro T, Kimura T, Morimoto T, Miyauchi K, Nakagawa Y, Yamagishi M (2009). Effect of intensive statin therapy on regression of coronary atherosclerosis in patients with acute coronary syndrome: a multicenter randomized trial evaluated by volumetric intravascular ultrasound using pitavastatin versus atorvastatin (JAPAN-ACS [JAPAN assessment of pitavastatin and atorvastatin in acute coronary syndrome] study). J Am Coll Cardiol.

[46] Tang X, Yang Y, Luo S, Zhao Y, Lu C, Luo Y (2016). The effect of statin therapy on plaque regression following acute coronary syndrome: a meta-analysis of prospective trials. Coron Artery Dis.

