# Supplementary figures and images for: High-intensity statin therapy yields better outcomes in acute coronary syndrome patients: a meta-analysis involving 26,497 patients
Source: Lipids Health Dis. 2020 Aug 23;19:194. doi: 10.1186/s12944-020-01369-6 (PMC7444068; doi:10.1186/s12944-020-01369-6)

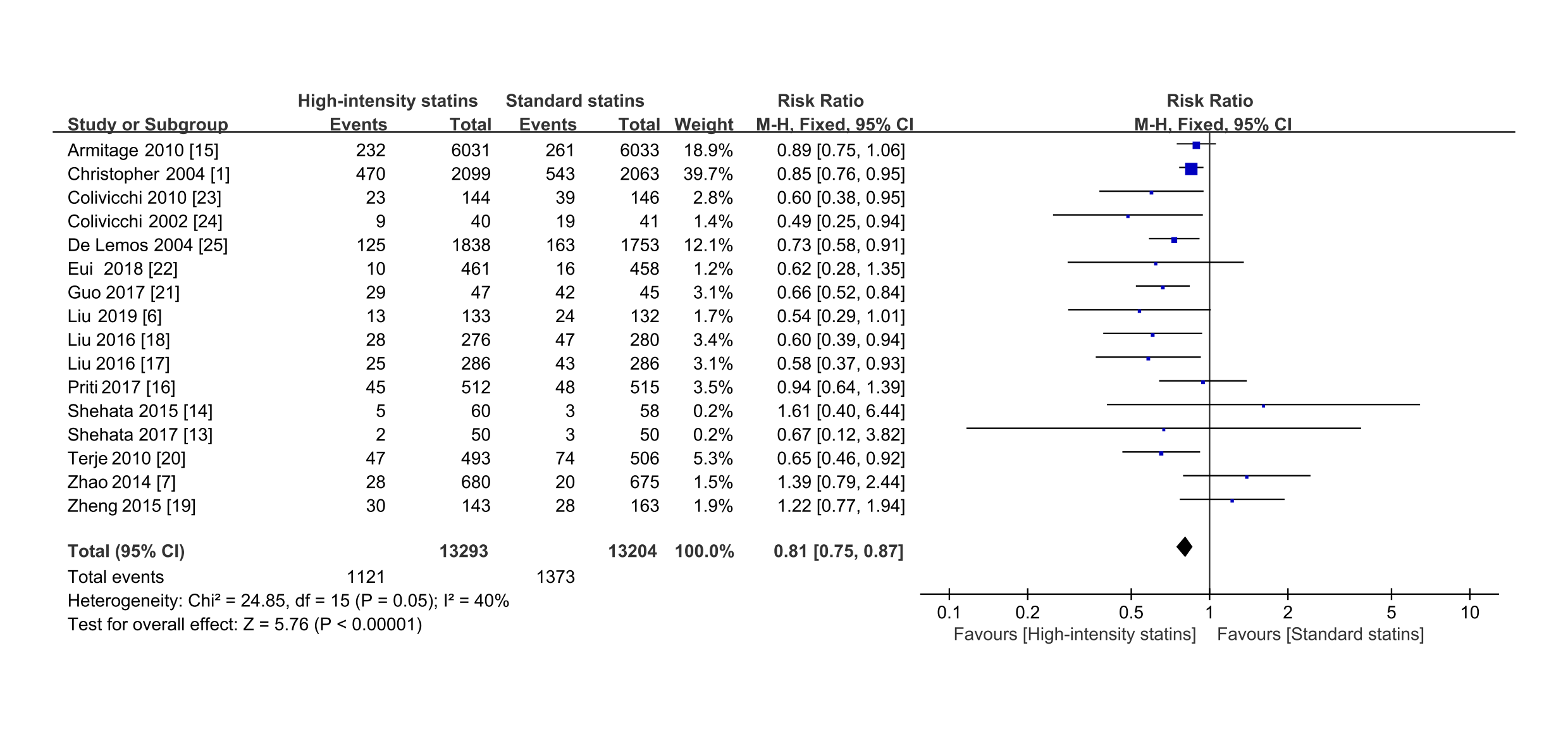

Supplement: Supplementary file 1 — Additional file 1: Figure S1. Forest plot of MACE, a) M-H for the fixed-effect model; b) Peto method for the fixed-effect model. RR, risk ratio; M-H, Mantel-Haenszel method; MACE, major adverse cardiovascular events. [file 12944_2020_1369_MOESM1_ESM.zip › Sfig1a.tif]

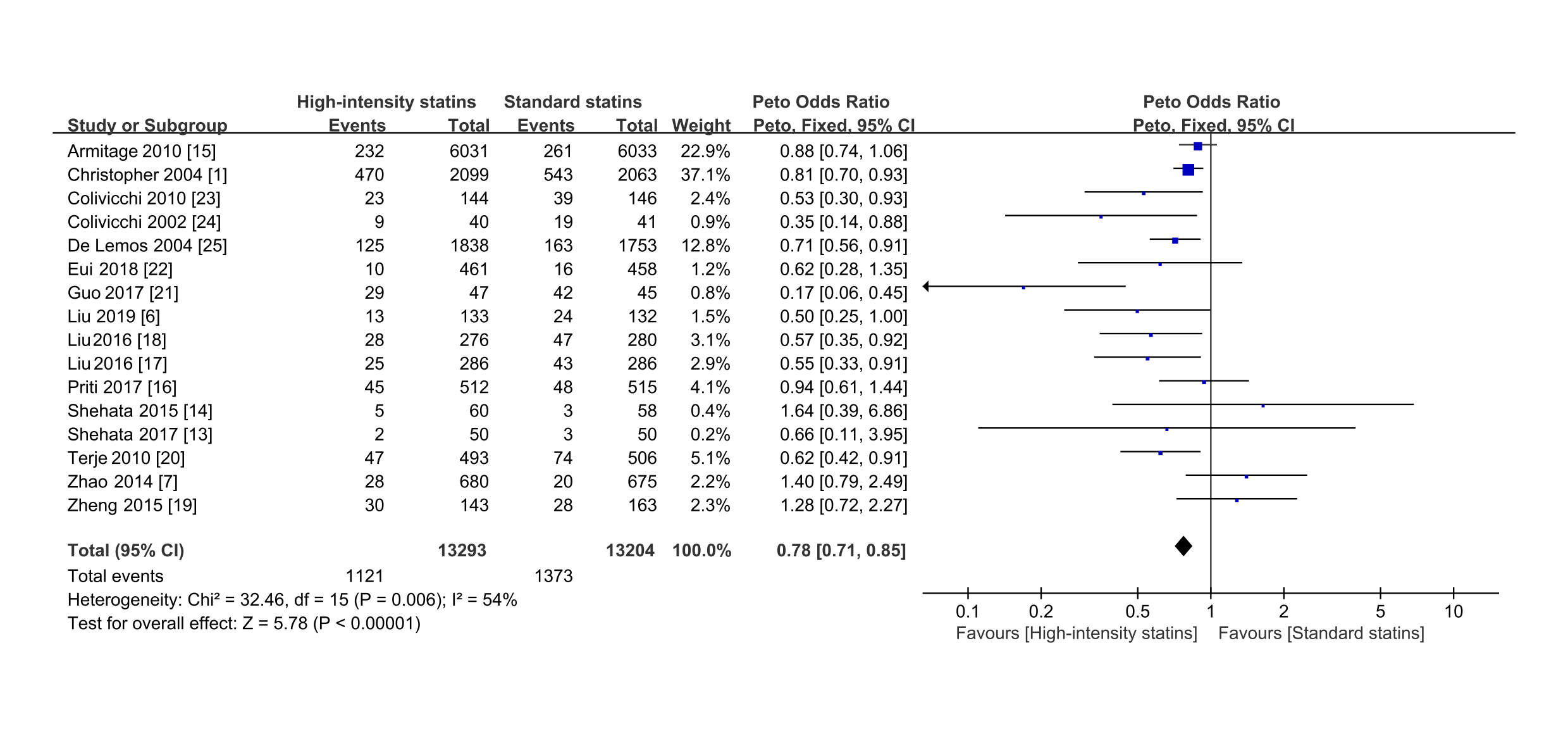

Supplement: Supplementary file 1 — Additional file 1: Figure S1. Forest plot of MACE, a) M-H for the fixed-effect model; b) Peto method for the fixed-effect model. RR, risk ratio; M-H, Mantel-Haenszel method; MACE, major adverse cardiovascular events. [file 12944_2020_1369_MOESM1_ESM.zip › Sfig1b.tif]

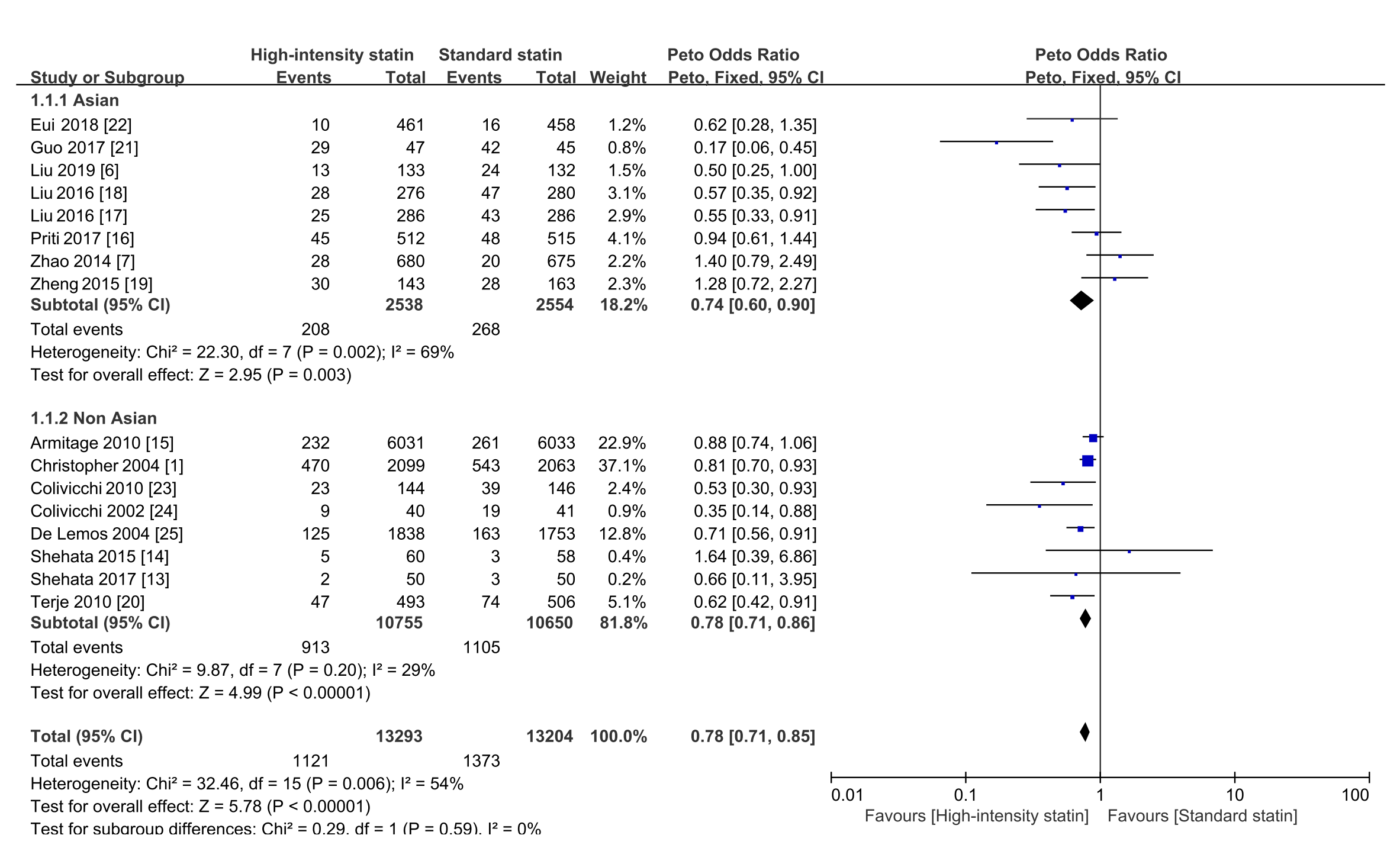

Supplement: Supplementary file 3 — Additional file 3: Figure S3. Forest plot of MACE by patient race. RR, risk ratio; MACE, major adverse cardiovascular events. [file 12944_2020_1369_MOESM3_ESM.tif]

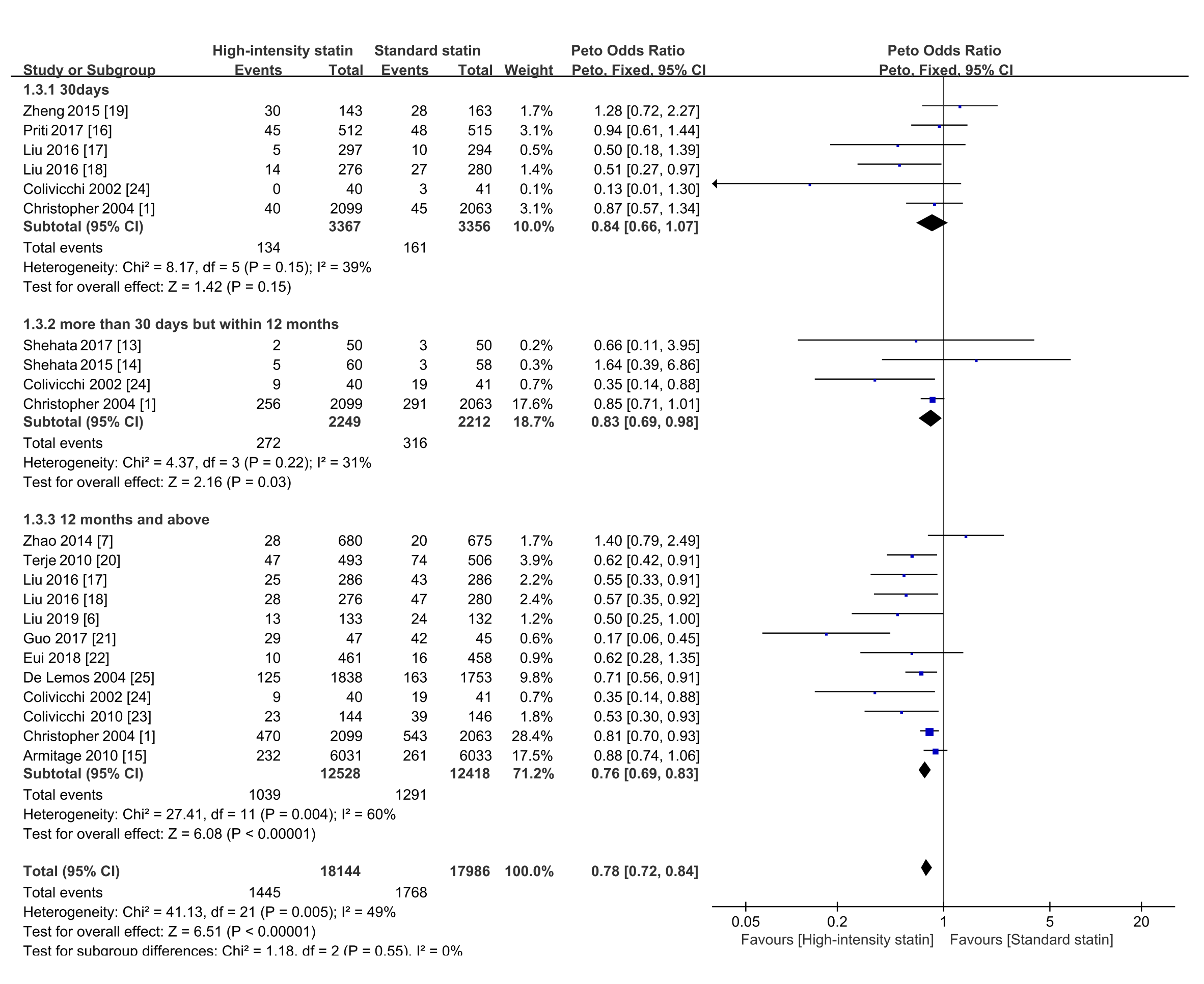

Supplement: Supplementary file 4 — Additional file 4: Figure S4. Forest plot of MACE by the duration of treatment. RR, risk ratio; MACE, major adverse cardiovascular events. [file 12944_2020_1369_MOESM4_ESM.tif]

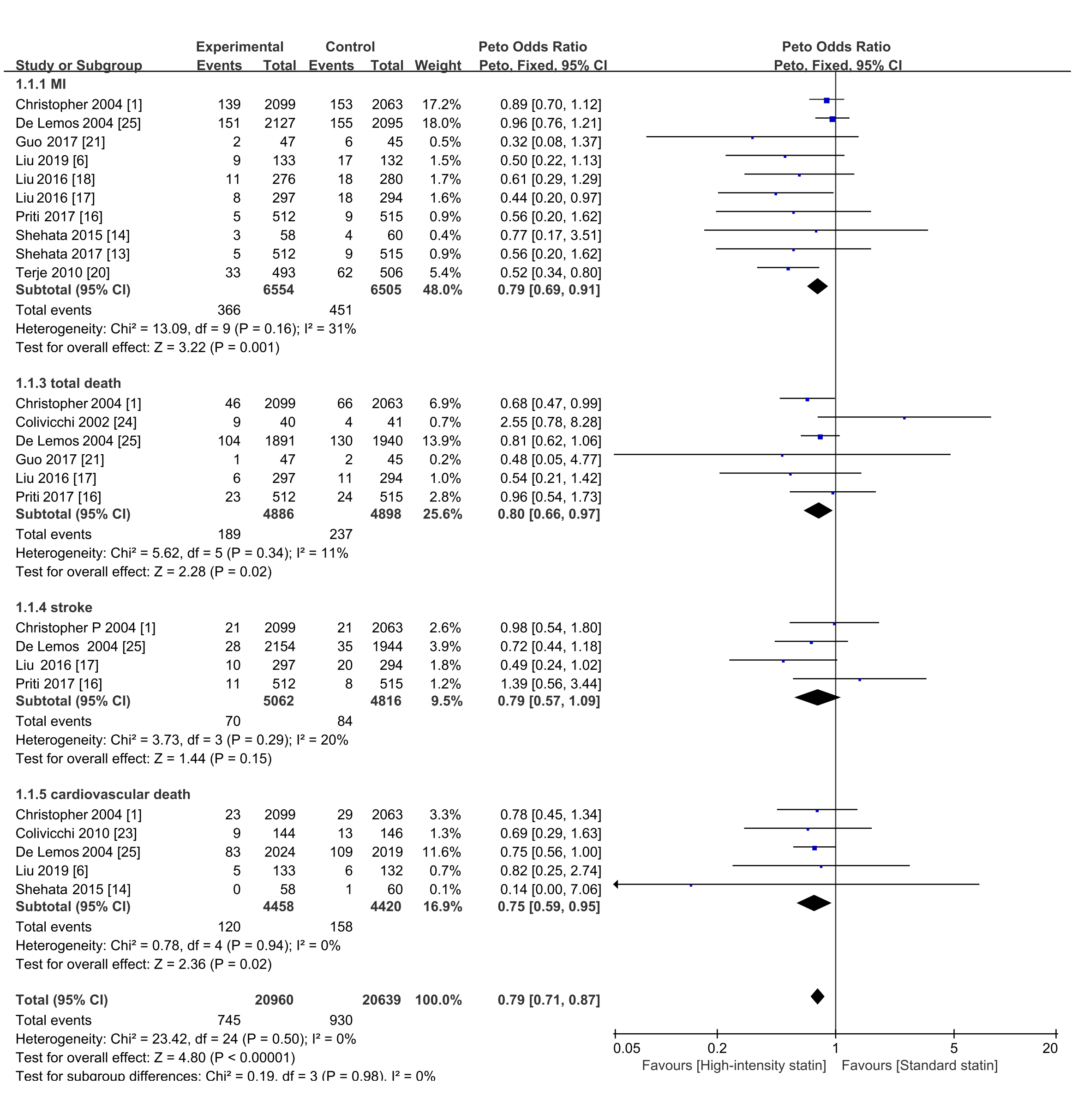

Supplement: Supplementary file 5 — Additional file 5: Figure S5. Forest plot of the secondary outcomes. RR, risk ratio. [file 12944_2020_1369_MOESM5_ESM.tif]

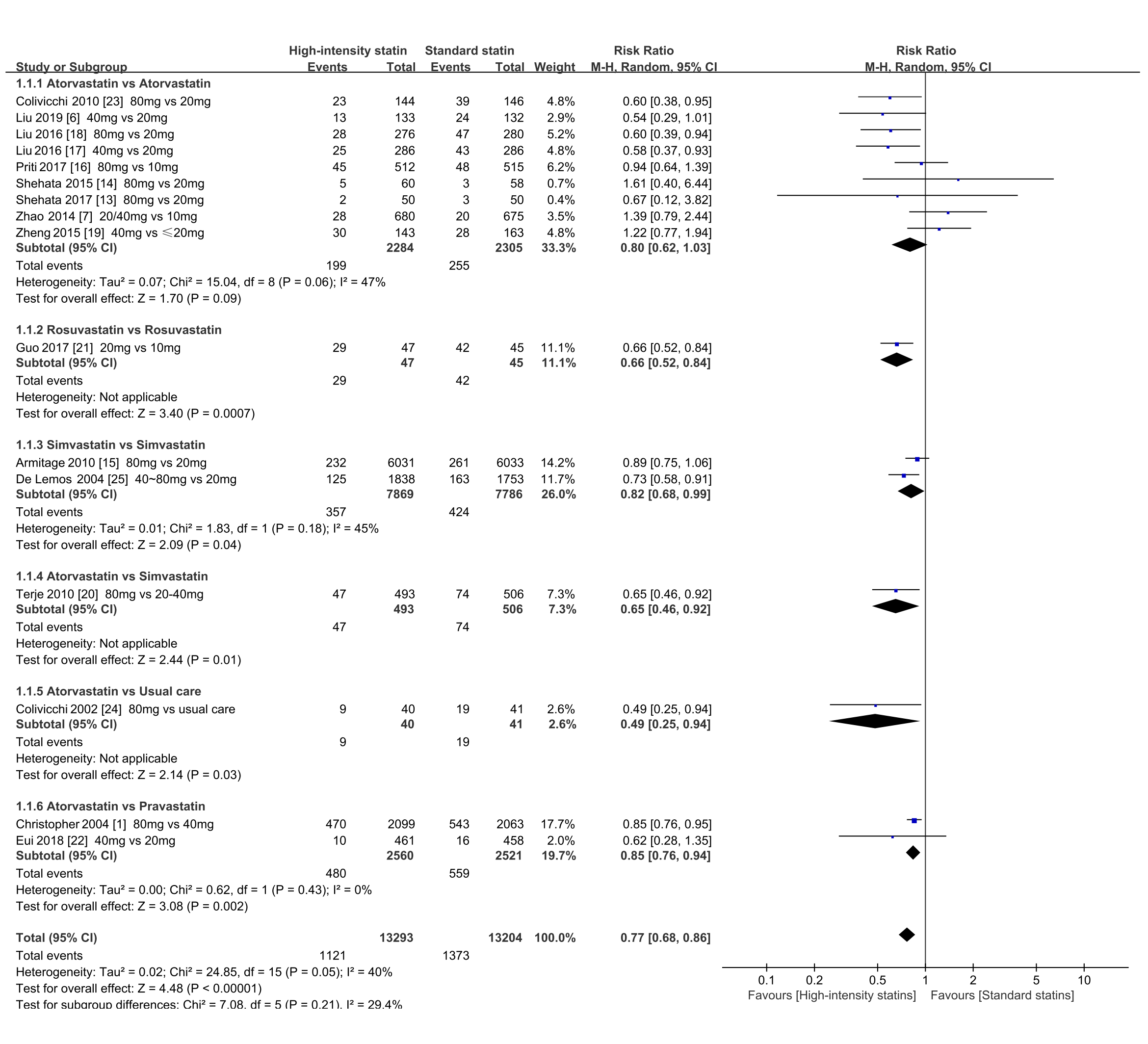

Supplement: Supplementary file 6 — Additional file 6: Figure S6. Differential effects of different statin regimens in the subgroup analysis. [file 12944_2020_1369_MOESM6_ESM.tif]
